# Variation in Gene Expression in Autism Spectrum Disorders: An Extensive Review of Transcriptomic Studies

**DOI:** 10.3389/fnins.2016.00601

**Published:** 2017-01-05

**Authors:** Ashley Ansel, Joshua P. Rosenzweig, Philip D. Zisman, Michal Melamed, Benjamin Gesundheit

**Affiliations:** Cell-El Ltd.Jerusalem, Israel

**Keywords:** autism spectrum disorders (ASD), gene expression, immune system, lymphoblastoid cell lines, monozygotic twins, Fragile X Syndrome, neurogenesis and inflammation

## Abstract

Autism spectrum disorders (ASDs) are a group of complex neurodevelopmental conditions that present in early childhood and have a current estimated prevalence of about 1 in 68 US children, 1 in 42 boys. ASDs are heterogeneous, and arise from epigenetic, genetic and environmental origins, yet, the exact etiology of ASDs still remains unknown. Individuals with ASDs are characterized by having deficits in social interaction, impaired communication and a range of stereotyped and repetitive behaviors. Currently, a diagnosis of ASD is based solely on behavioral assessments and phenotype. Hundreds of diverse ASD susceptibility genes have been identified, yet none of the mutations found account for more than a small subset of autism cases. Therefore, a genetic diagnosis is not yet possible for the majority of the ASD population. The susceptibility genes that have been identified are involved in a wide and varied range of biological functions. Since the genetics of ASDs is so diverse, information on genome function as provided by transcriptomic data is essential to further our understanding. Gene expression studies have been extremely useful in comparing groups of individuals with ASD and control samples in order to measure which genes (or group of genes) are dysregulated in the ASD group. Transcriptomic studies are essential as a key link between measuring protein levels and analyzing genetic information. This review of recent autism gene expression studies highlights genes that are expressed in the brain, immune system, and processes such as cell metabolism and embryology. Various biological processes have been shown to be implicated with ASD individuals as well as differences in gene expression levels between different types of biological tissues. Some studies use gene expression to attempt to separate autism into different subtypes. An updated list of genes shown to be significantly dysregulated in individuals with autism from all recent ASD expression studies will help further research isolate any patterns useful for diagnosis or understanding the mechanisms involved. The functional relevance of transcriptomic studies as a method of classifying and diagnosing ASD cannot be underestimated despite the possible limitations of transcriptomic studies.

## Background

Autism spectrum disorders (ASDs) are a group of complex neurodevelopmental conditions that present in early childhood. Individuals with ASDs are characterized by having deficits in social interaction, impaired communication and a range of stereotyped and repetitive behaviors (Lord et al., 1994). In 2012, ASD has a current estimated prevalence of about 1 in 68 US children aged 8 years; estimated prevalence was significantly higher among boys (23.6 per 1000) than among girls (5.3 per 1000) (Christensen et al., 2016). ASDs are heterogeneous, and arise from epigenetic, genetic and environmental origins. The exact etiology of ASDs still remains unknown and ASD cases with a genetic etiology only collectively account for 10–20% at most (Abrahams and Geschwind, 2008). The precise role of genetics in the pathogenesis of ASD remains unclear. On the one hand, there are several hints that genetics play a role (Wang et al., 2009). For example, there are much higher concordance rates of ASDs in monozygotic twins (70–90%) than dizygotic twins (0–10%) (Abrahams and Geschwind, 2008). Similarly, the recurrence risk in families ranges from 12.9% (Sandin and Reichenberg, 2014) to 18.7% (Ozonoff et al., 2011; Yuen et al., 2015). Furthermore, more than 100 ASD-susceptibility genes have already been identified (Yuen et al., 2015). On the other hand, these specific genetic mutations account for less than 8% of cases (Alter et al., 2011). There are a number of explanations for this ambiguity ranging from gene-gene interactions, to the heterogeneity of the disease, to epigenetic factors (Alter et al., 2011). Broad gene expression screening of children diagnosed with ASD is one approach toward mitigating the challenge of the heterogeneity of ASD by separating those diagnosed with ASD into subclasses according to gene expression profiles.

In the most recent comprehensive review of gene expression studies in ASD in 2012, Voineagu et al. surveyed 10 major mRNA studies across two types of samples [lymphoblastic (LBL) and post-mortem brain tissue]. They found that transcriptome analysis was more efficient than DNA studies in identifying differences between ASD and controls. Focusing on human mRNA studies since 2011 in English that monitor up or down regulation of multiple genes in ASD, without comparing to other disorders, we find that 27 major new studies have been published. By considering both single gene studies and more complex studies looking at pathways, researchers will be able to compare their findings to individual lists of genes but also to attempt to place their findings in a broader context by looking at pathway networks implicated in ASD. For example, if a researcher identifies a single specific gene to be upregulated in a specific type of tissue in ASD, that can then be compared to other single gene studies across different tissue types to see if there is consistency in the directionality of regulation and across tissue types. Additionally, by looking at a variety of networks and pathways, researchers can see the interaction between various neurodevelopmental and immune processes. These studies follow similar methodology to the studies previously discussed; though some expand to a third tissue type namely, intestinal biopsy samples.

## Gene expression studies in ASD by tissue type

In reviewing the new gene expression studies since Voineagu's paper (Table 1), the classification according to sample source will prove helpful. Five sample sources were studied: post-mortem brain, peripheral blood, gastrointestinal tissue, adult olfactory stem cells, and scalp hair follicles. Before beginning with the post-mortem brain tissue studies, it is relevant to mention gross pathological and radiological brain findings that might provide context for the brain-related expression findings.

**Table 1:** **Gene Expression Studies of Autism: 2011 onwards (Year chosen since Voineagu et al. review paper is from 2012)**.

**Study**	**Number of samples**	**Tissue source**	**Number of dysregulated genes listed**
Alter et al., 2011	82 children with autism (mean: 5.5 years SD 2.1 *p* < 0.0001) and 64 controls (mean: 7.9 years SD 2.2 *p* < 0.0001)	PBL	Children w/autism/Children w/younger fathers: 2093 significantly downregulated & 641 significantly upregulated genes; Children w/older fathers: Children w/younger fathers: 1476 significantly downregulated & 764 significantly upregulated genes; 593 genes were downregulated and 145 genes were upregulated in both children with autism and children of with older fathers
Anitha et al., 2012	9 autism patients ant 8 controls	ACG, MC, THL	28 genes showed brain region-specific reduced expression in autism
Chien et al., 2013	PART I: Conducted comparative total gene expression profiling analysis between 16 male patients with ASD (age range 4–18 years) and 16 male control subjects (age range 18–67 years); PART II: compared transcript level of one particular gene (FOXP1) between 83 male patients with ASD and 83 male healthy controls	LCL derived from the EBV transformation of lymphocytes of peripheral blood	202 genes were differentially expressed in the ASD group, including 89 upregulated and 113 downregulated
Chow et al., 2012	16 from young postmortem males (2–14 years; autism = 9, control = 7) and 17 adult males (15–56 years, autism = 6, control = 11)	DLPFC	2017 genes across all autistic and control cases independent of age
Féron et al., 2016	9 adults with severe autism and low to very low developmental disabilities, plus two adults with mild or moderate autism and no or mild cognitive abilities (Asperger syndrome or high functioning autism) paired with 11 age and gender matched controls	Adult nasal olfactory stem cells	156 genes that were differentially expressed in at least one ASD patient, of which 31 were dysregulated in more than a third of the cohort
Ginsberg et al., 2012	9 autism and 9 control subjects	BA19 (occipital) brain tissues	876 unique, annotated genes differently expressed between autistic and control brains
Glatt et al., 2012	60 infants and toddlers at risk for ASDs (autistic disorder and pervasive developmental disorder), 34 at risk for LD, 17 at risk for DD, and 68 TD children	PBMCs	154 probes showed significant dysregulation in ASD
Chana et al., 2015	Utilized published microarray data from 30 control and 27 ASD individuals	DLPFC	3 downregulated genes and 1 upregulated gene in ASD samples
Ivanov et al., 2015	30 subjects with idiopathic autism (24 male, 6 female) aged 3 to 11 years (mean age of sample 6.86 years) and 30 healthy children age and sex matched	Peripheral blood	23 differentially expressed genes (10 upregulated, 13 downregulated)
James et al., 2014	13 autism and 13 unaffected control individuals	Cerebellar cortex	7 genes
Khan et al., 2014	11 control (4F,7M) and 10 ASD (3F,7M) cases were examined (Controls ranged between 5 and 16 years of age, while ASD ranged from 4 to 15 years of age)	CB, BST, CG, ORC, PT, Wer	15 genes showed brain-region specific dysregulated expression in ASD samples
Kong et al., 2013	20 proband-unaffected sibling pairs (5 probands-sib pairs were of the same gender, i.e., males, while 15 pairs were of the opposite gender including 12 male and 3 female probands) and 18 unrelated control (11 males, 7 females) individuals	Peripheral blood	163 unique genes were significantly changed between probands and siblings
Kuwano et al., 2011	GROUP 1: 21 Young adults with ASD (17 males and 4 females) aged 26.7 ± 5.5 years, age range: 18-38 years; GROUP 2: 21 age and gender matched healthy controls aged 27.0 ± 5.5 years, age range: 19–39; GROUP 3: 21 Healthy mothers having children with ASD (asdMO), aged 44.7 ± 6.7 years, age range: 33–58 years; GROUP 4: asdMO control, aged 44.7 ± 6.7 years, age range: 31–59 years	Peripheral blood	ASD/control: 19 genes were found to be significantly dysregulated (18 upregulated and 1 downregulated); asdMO/asdMO control: 57 genes were found to be significantly dysregulated (17 upregulated and 40 downregulated); 3 genes overlapped and were dysregulated both in individuals with ASD and in asdMO
Maekawa et al., 2015	24 male control subjects (Aged 32.60 ± 3.91) and 18 Autism subjects (16 male, 2 female; aged 25.61 ± 4.95)	Scalp hair follicles	1 gene
Prandini et al., 2014	Two separate series of sib-pairs totaling 36 children and adolescents between 4 and 18 years of age	LCLs	none found
Segura et al., 2015	21 adolescents and adults diagnosed as ASD (20 males, 1 female) as well as from 10 healthy controls (10 males)	Whole blood	3 genes
Stamova et al., 2011	33 boys with AU (mean age 45.3 months; age range of 31–60 months) and 51 age-matched TD control boys (mean age 43.3 months; age range 28–57 months)	Whole blood	11 genes
Talebizadeh et al., 2014	Autistic group included three females (6, 11, and 13 years old) and two males (5 and 12 years old) diagnosed with classical autism and 5 age and sex matched unrelated controls	LCL derived RNAs	57 genes
Taurines et al., 2011	51 children with ADHD, 26 children with ASD (19/26 comorbid with ADHD) and 39 TD	Whole blood cells	2 genes
Tian et al., 2011	37 children with autism (32 males, 5 females; average age 44.2 ± 10 months) compared to 15 typically developing controls (11 males, 4 females; average age 41.2 ± 6 months)	Whole blood	31 genes
Voineagu and Eapen, 2013	19 autism cases and 17 controls	STG, prefrontal cortex (BA9) and cerebellar vermis	444 genes showing significant expression changes in autism cortex samples, 2 genes differentially expressed in cerebellum
Walker et al., 2013	25 children with a diagnosis of ASD (mean age 5.0862.06 years; 23 male and 2 female, 16 had a diagnosis of autism; 9 had a diagnosis of autism spectrum disorder); 3 TD groups: (1) 15 children with no chronic GI symptoms (mean age 12.263.07 years; 6 male and 9 female); (2) children with a diagnosis of Crohn's disease (*n* = 8, mean age 12.9763.07 years; 3 male and 5 female); (3) children with a diagnosis of ulcerative colitis (*n* = 5, mean age 12.064.0 years; all female)	Tissue specimen from 7 anatomic locations (from terminal ileum to rectum)	Ileal mucosa: ASD-GI/TD -1409 differentially expressed transcripts • Colonic mucosa: ASD-GI/TD -1189 differentially expressed transcripts: Overlap between both sets ASD-GI (Ileum and Colon)/ TD -178 transcripts exclusively differentially-expressed
Williams et al., 2011	15 AUT-GI children (mean onset age 13.4+/25.4 months, median age at biopsy 4.5), 7 Control-GI patients (median age at biopsy 4.0)	Ileum and Cecum	6 genes
Yasuda et al., 2011	35 patients with ASD (mean age 12.9 years ± 12.4 SD) 35 healthy controls (mean age 34.8 years ± 9.7 SD)	LCLs	2 genes
Zhubi et al., 2014	10 ASD and 10 control samples	Cerebellar cortex	4 genes
Ziats and Rennert, 2013	Re-analyzed sex-specific gene-expression from a recent large transcriptomic study (Kang et al., 2011); *N* = 57, including 39 with both hemispheres; age, 5.7 post-conceptual weeks to 82 years; sex, 31 males and 26 females;	Transient prenatal structures and immature and mature forms of 16 brain regions (Kang et al., 2011). Table 2)	37 Female and 123 Male genes found to be differentially expressed by sex, and their brain region and developmental time point

*PBL, Peripheral blood lymphocytes; LCL, lymphoblast cell line; PBMCs, peripheral blood mononuclear cells*.

*ACG, anterior cingulate gyrus; MC, motor cortex; THL, thalamus; DLPFC, Dorsolateral Prefrontal Cortex; CB, Cerebellar; BST, Brain stem*.

*CG, Cingulated gyrus; ORC, Orbitofrontal cortex; PT, Putamen; Wer, Wernicke's; STG, superior temporal gyrus*.

*LD, language delay; DD, global developmental delay; TD, typically developing*.

### Neurological background

Evidence for neurological involvement in ASD can be divided into neuroimaging and post-mortem pathology.

#### Neuroimaging

Neuroimaging studies of children with autism have revealed abnormal brain overgrowth in prefrontal, temporal and amygdala regions and abnormal functional asymmetry and activation in the cortex and cerebellum (Chow et al., 2012). Neuroimaging techniques such as functional MRI has shown altered patterns of functional specialization in autism in several domains of thinking, such as cognitive, linguistic, social, and visuospatial processing in children and adults with ASD (Maximo et al., 2014). Impaired functional connectivity may demonstrate inefficiency in maximizing network connections to execute tasks, yet the findings of over-connectivity have been interpreted to reflect hyper-specialized, rather than more efficient connectivity. Based on the abundance of information, inefficient connectivity may be the hallmark of ASD.

#### Pathology

Pathology on postmortem brains from subjects diagnosed with ASD have demonstrated abnormalities in neuronal organization of the cerebral cortex and decreased number of Purkinje cells in the cerebellum (Garbett et al., 2008). Children between 2 and 4 years old diagnosed with ASD have been found to have increased total cerebral gray and white matter, excess neurons in the pre-fontal cortex, and an increase in brain weight at autopsy (Courchesne et al., 2011; Hazlett et al., 2011). In some children diagnosed with ASD, the rate of head growth and brain growth is exceptionally rapid during the first few years of life. Additionally, although most children with autism are born normocephalic, during the first years of life, 15–20% will develop macrocephaly (Lainhart et al., 2006). However, by late adolescence and early adulthood, the autistic brain commonly displays neuron loss and cortical thinning and is no longer enlarged (Kates et al., 2004).

## Post-mortem brain tissue gene expression studies

With the background provided by the neuroimaging and pathology findings in mind, gene expression studies in brain tissue can now be reviewed. There are nine gene expression studies that look at a variety of brain regions. The studies are all very small but produced large numbers of genes with variable expression between ASD and control. Chow et al. analyzed frozen samples of dorsolateral prefrontal cortex from 16 young postmortem males (9 autism, 7 control) and 17 adult males (6 autism, 11 control) in order to study age-dependent brain gene expression (Chow et al., 2012). They found 102 genes, which contrasted between young children diagnosed with ASD and control groups, and 736 genes, which contrasted between adults diagnosed with ASD and control groups. Ginsberg et al. analyzed cerebellar and BA19 (Brodmann Area 19, occipital) brain tissues from 9 control and 9 autism patients (Ginsberg et al., 2012). After correcting for the region of the brain, they discovered 876 unique, annotated genes expressed differently between autistic and control brains. This was a false discovery rate of five percent. Anitha et al. compared the expression of 84 electron transport chain genes belonging to the 5 complexes in the post-mortem brains of 8 autism patients and 10 controls. They found that 11 genes of Complex I, 5 genes each of Complex III and Complex IV, and 7 genes of Complex V (28 genes in total) showed brain region-specific reduced expression in autism (Anitha et al., 2012). Voineagu et al. profiled post-mortem samples of the superior temporal gyrus, prefrontal cortex, and cerebellar vermis regions from 19 autism and 17 control cases (Voineagu and Eapen, 2013). They identified 444 genes, which showed significant expression changes in autism cortex samples as compared to the controls, while only 2 genes were differentially expressed in the cerebellum. Ziats et al. re-analyzed a large transcriptomic study in the developing brain that was done by (Kang et al., 2011; Ziats and Rennert, 2013). The study performed genome-wide microarray analysis on postmortem human brain tissues from 16 brain regions spanning preconception to adulthood, specifically looking to identify sex-biased gene expression in the developing brain. Thirty-seven female, and 123 male genes were found to be differentially expressed by sex, brain region, and developmental time point. Khan et al. analyzed altered thyroid hormone-dependent brain gene expression by studying various brain regions from 10 ASD and 11 control cases (Khan et al., 2014). They found 14 genes that were differentially expressed based on sex-specific brain area. For example, expression of the DIO2 gene, a gene involved in thyroid hormone activation, was increased in the putamen (*p* < 0.05) and there was a trend toward increase in cingulated gyrus (p = 0.08) of the female ASD cases, while expression of the Cirbp gene, a gene responsible for stabilizing transcripts of genes involved in cell survival, was decreased (*p* < 0.05) in the putamen of ASD male cases only. James et al. compared 1 sq cm blocks of cerebellar cortex from 13 individuals with ASD and 13 controls (James et al., 2014). They observed a significant increase in both 5-mC and 5-hmC in the cerebellum of individuals with ASD relative to control samples. DNMT3A, DNMT3B, TET1, TET3, which are all genes related to methylation and 8-oxo-deoxyguanosine (8-oxo-dG) (a major product of DNA oxidation) expression levels were significantly increased in the cerebellum of individuals with ASD relative to control samples. They also found that within the EN-2 promoter sequence there was a statistically significant positive association between 5-hmC (a gene important in epigenetics) and EN-2 gene expression. Additionally an association between 5-hmC and EN-2 gene expression in the 5′ promoter CpG Island was found in people diagnosed with ASD but not in controls. Studies of MeCP2 (a chromosomal protein that binds to methylated DNA) binding in the EN-2 promoter demonstrated a significant decrease in repressive MeCP2 binding to the identical 5′ promoter region that contained increased levels of 5-hmC in individuals with ASD relative to control samples. Zhubi et al. also compared blocks of cerebellar cortex from 10 individuals with ASD and 10 control samples. They found a one and a half to two time increase in binding of MeCP2 to GAD1 and RELN promoters in the cerebellum of individuals diagnosed with autism when contrasted to controls. RELN plays a role in layering of neurons in the cerebral cortex and cerebellum. They detected that levels of 5-hmC were significantly enriched at GAD1 and RELN promoters in individuals with ASD. The methylation changes they found lead to a remarkable increase in the amounts of 5-hmC relative to 5-mC. The 5-hmC/5-mC ratio at the GAD1 promoter is 5.5 in people diagnosed with ASD and only 1.2 in controls. Similar to James et al. (2014), they also found TET1 mRNA levels to be increased in the cerebella of individuals with ASD (Zhubi et al., 2014). Chana et al. (2015) compared data from brain tissue from 30 controls and 27 individuals diagnosed with ASD. They found that expression of mGluR5 was decreased in ASD when compared to controls. This gene has been found to be associated with the forming of synapses, activation of microglia, and other processes.

### Immune system

One study demonstrated findings in post-mortem brain tissue that were specific to the immune system. Considering the extensive literature analyzing the relationship between the immune system and ASD (Gesundheit et al., 2013), this demands further attention. Garbett et al. analyzed frozen samples of superior temporal gyrus from 6 subjects with ASD and 6 controls (Garbett et al., 2008). Based on four parameters, they identified 152 differentially expressed gene products, and of these, 130 demonstrated increased expression while 22 showed decreased levels in the brains of individuals with ASD. Seventy-two annotated differentially expressed transcripts were either cytokine responsive transcripts or transcripts related to the immune system. Additionally, they noticed decreased transcript levels for a number of genes involved in outgrowth and neuronal differentiation. They used Gene Set Enrichment Analysis, which marks functional pathways in which gene expression changes are grouped together. This enabled the identification of 31 gene sets that were differentially expressed between individuals with autism and control samples. Of the 31, 19 genes were involved in immune system function.

## Peripheral blood gene expression studies

The second and most common source used in gene expression studies for autism was lymphoblastoid cell lines (LCLs). There is an apparent discrepancy between neuroanatomical and cellular abnormalities observed for autism at younger ages and molecular pathologies at more advanced ages. Hu et al. analyzed LCLs derived from lymphocytes of 3 pairs of monozygotic twins that were discordant with respect to clinical diagnosis of ASD (Hu et al., 2006). Twelve hundred genes were identified as significant with a false discovery rate of 26%, 25 genes were found to be up-regulated at least 1.5-fold in the more severely affected twin relative to the other twin (log2 (ratio) = 0.58) and 19 genes were down-regulated by at least 1.5-fold. Of these, eight of the 26 genes match genes connected to neurological function, development, or disease. In 2009, Hu et al. conducted microarray analysis on 116 LCLs from individuals with idiopathic autism who were separated into three phenotypic subcategories according to severity scores from the ADI-R questionnaire and age-matched, typically developing controls. They identified 530 significantly differentially expressed genes that distinguished controls from all samples with ASD. Hu et al. analyzed gene expression profiling and how it differentiates ASD from controls and phenotypic variants of ASD (Hu et al., 2009). Microarray analyses were conducted on 116 LCLs from individuals with idiopathic ASD who were separated into three phenotypic subgroups according to severity scores from the ADI-R questionnaire and age-matched, non-autistic controls. Five hundred and thirty significantly differentially expressed genes were found that distinguished the samples of ASD from controls. They also identified 123 significant genes from 4-class significance analysis of microarrays (SAM) analysis of data from gene expression from severe, mild, and savant subgroups and the non-autistic control groups. When the pathways of the overlapping genes were analyzed between the severe language and mild autism subgroups, a network of genes that affect common functional targets, such as synaptic transmission, neurogenesis, neurulation, long-term potentiation (learning), protein ubiquitination, and brain function was revealed. Additionally, 15 significant differentially expressed genes that regulate circadian rhythm were found, unique to the most severely affected ASD subgroup. Differential expression of these genes was observed only in the samples from severely language-impaired individuals (L subgroup), with each individual showing altered expression of multiple (but not all 15) genes. Emanuele et al. investigated increased dopamine DRD4 receptor mRNA expression in lymphocytes of individuals diagnosed with autism and musicians in order to explore the music-autism connection. The DRD4 receptor is known to be responsible for neuronal signaling in the mesolimbic system of the brain. They studied 20 ASD patients, 19 professional adult musicians, and 19 gender and age matched control individuals who were not interested in playing or listening to music. Analysis of variance (ANOVA) demonstrated significant differences in DRD4 mRNA expression between the groups (*P* = 0.008). *Post-hoc* analysis highlighted significant differences between the control group and both musicians (*P* < 0.05) and individuals diagnosed with ASD (*P* < 0.05) (Emanuele et al., 2010). Yasuda et al. measured mRNA expression levels in lymphoblastoid cells from 35 subjects with ASD and 35 controls. They found that in individuals diagnosed with autism, B-actin normalized NLGN3 expression levels or TATA-binding protein were decreased by 35 or 26% respectively. They also found that gene expression levels of the SHANK3 gene, a gene that codes for a major scaffold postsynaptic density protein, regulated by B-actin or TATA binding protein were also decreased in individuals with autism by 39 or 40% respectively (Yasuda et al., 2011).

Stamova et al. looked for correlations between gene expression and mercury level in blood of boys with and without ASD. They collected whole blood from 33 boys with ASD and 51 age-matched typically developed (TD) control boys. There was no significant difference in Hg levels between the ASD and TD groups. They found 11 genes whose expression correlated inversely with mercury levels in boys diagnosed with autism compared to typically developing children. One limitation of this study was that samples collected from children ages 2–5 do not consider the possible direct role of mercury as a causal factor for autism, which likely starts *in utero* or shortly after birth (Stamova et al., 2011).

Tian et al. looked for correlations of gene expression with blood lead levels in children diagnosed with autism contrasted to typically developed controls. They looked at 37 children with ASD, and 15 TD controls. There was no significant difference in blood lead levels. Forty-eight probe sets represent 31 genes, whose expression correlated with lead levels in each group, and the partial correlation coefficients were statistically different between the groups; most of the genes are negatively correlated with blood lead levels in typically developed children and positively correlated with blood lead levels in children diagnosed with autism. The conclusion of their study, however was that lead most likely does not explain the increasing incidence in ASD (Tian et al., 2011).

Alter et al. looked at ASD and changes related to paternal age in overall levels of gene expression regulation. They performed gene expression microarrays on RNA from peripheral blood lymphocytes of 82 children with ASD and 64 controls where parental age was similar between the 2 groups. They then performed a secondary analysis by analyzing paternal age difference as a risk factor for ASD and whether it was associated with variance. They found that the distribution of gene expression levels on microarrays from individuals with autism had a decreased variance when compared to microarrays from controls (*p* = 0.006). They also found that in controls, but not in children with ASD, overall variance in gene expression was found to be significantly and negatively associated with the age of the father (*p* = 0.03), so as predicted, the overall variance was the same in children of fathers who were older and children with ASD with fathers of any age. In the comparison of children with ASD to children with fathers of a younger age, there were 2093 genes that were significantly downregulated by at least 1.1-fold, and only 641 that were upregulated. In the blood of children with fathers who were older compared to the children with younger paternal age, there were 1476 downregulated and 764 upregulated genes. There were 593 genes that were downregulated in both children with ASD and children of with older fathers (*p* < 0.000001) and 145 genes that were upregulated in both comparisons (Alter et al., 2011).

Chien et al. looked at increased gene expression of FOXP1 by comparing LCL between 16 males diagnosed with ASD and 16 male controls. FOXP1 is a transcriptional repressor. A total of 252 differentially expressed probe sets corresponding to 202 genes were detected between the 2 groups, including 89 up- and 113 down-regulated genes in the group diagnosed with autism. Real-time quantitative polymerase chain reaction (RT-qPCR) verified significant elevation (1.89 ± 2.64, *P* = 0.005) of the FOXP1 gene transcript of LCL in a sample of 83 male patients, compared with 83 male controls. Using three platforms, they found several immune-related pathways showing significant differences between the ASD patients and controls (Chien et al., 2013).

Prandini et al. analyzed RBFOX1 gene expression in LCL of Italian discordant ASDs sib-pairs totaling 36 children and adolescents. Their data showed however, that RBFOX1 normalized mean values were not significantly different between controls and those diagnosed with ASD, they suggested a possible cause for this might have been due more subtle transcription level differences in RBFOX1 gene expression in LCL than in brain samples (Prandini et al., 2014).

Talebizadeh et al. performed a pilot study and looked at exon-level expression profiling and alternative splicing in ASD using LCL. They found 57 genes that were differentially expressed at the exon level between ASD and control samples. They also found differential splice variants of the gene CYFIP1 (which has 2 protein-coding transcripts in the literature), exon array analysis demonstrated a higher expression for the probe sets which binds to exon 16 in variant 1 (encoding a long form) in subjects with ASD vs. controls. DNA sequencing following RT-PCR for variant 1 also detected a product missing exon 16 inducing a premature stop codon, and qRT-PCR showed a higher expression of variant 1 in ASD compared with control samples. RT-PCR reactions were run for TRAP150 and ZMYM6 and DNA sequencing of the amplified products supported the fact that these exons undergo alternative splicing and enabled the indication of previously unreported alternative splicing isoforms for these two genes. These new variants included one isoform of TRAP150 (missing exon 4 resulting in an in-frame loss of 301 amino acid residues) and five alternatively spliced ZMYM6 variants that are labeled on the basis of the missing exons (isoforms missing exon 2, exon 4, exons 2&4, exons 2&5, and exons 2, 4, &5). ZMYM6 variants missing exon 2, the location of the start codon, most likely do not code for proteins and the exclusion of only exon 4 introduces a premature stop codon (Talebizadeh et al., 2014).

### Immune system-background

The role of the immune system in ASD is an active area of research. Evidence of an immune role in at least a subset of children diagnosed with ASD can be divided into brain antibodies, serum cytokines, family history and immunogenetics (Gesundheit et al., 2013).

### Immune system-gene expression studies

Gregg et al. subdivided the children into three groups based on various clinical criteria. Their sample size included 49 children on the autism spectrum and 12 controls. Unpaired *t*-tests detected a number of genes that were regulated more than 1.5-fold for autism vs. general population (*n* = 55 genes), for history of early onset vs. general population (*n* = 140 genes), and for developmental regression vs. general population (*n* = 20 genes). The three gene lists from the analysis were used to identify a small group of 11 genes that are shared between the three groups. These genes were all expressed in natural killer cells and many belonged to the KEGG natural killer cytotoxicity pathway. Database for Annotation, Visualization and Integrated Discovery (DAVID) and Ingenuity Pathway Analysis were used to analyze pathways, and notable pathway overlaps included natural killer cell signaling in all three comparisons, IL-2 signaling and serotonin receptor and dopamine receptor signaling in autism vs. general population and early onset vs. general population, and retinol and methionine metabolism in the early onset vs. general population analysis (Gregg et al., 2008).

Enstrom et al. analyzed peripheral blood from 35 children with ASD and 11 age and gender matched controls. They discovered that a total of 626 probes showed differential gene expression between the two groups (82 significantly higher and 544 significantly lower in the ASD group). The 82 upregulated probes in ASD correlated to 59 known genes, most of which have been connected to leucocyte function, more specifically the function of natural killer cells. Using microarray analysis, their studies demonstrated that 12 gene probes, corresponding to 11 different genes were differentially expressed in early onset and regressive types of ASD when compared with the control group. Flow cytometric analysis of natural killer cells demonstrated increased production of granzyme B, perforin, and interferon gamma (IFNγ) under resting conditions in children diagnosed with ASD (Enstrom et al., 2009).

Kuwano et al. looked at ASD-associated gene expression in peripheral leucocytes that were often noticed between subjects with ASD, and healthy mothers of children with ASD. They used DNA microarray to perform gene expression profiling in peripheral blood on 21 individuals from 4 groups: young adults with ASD, age and gender matched controls, mothers having children with ASD (asdMO), and age matched controls having healthy children. They found 19 genes that were significantly differentially expressed (18 up and 1 down-regulated) when comparing the ASD to the control group, and 57 genes that were differentially expressed between the asdMO group and the asdMO control group (17 up-regulated and 40 down-regulated genes fold change >2.0). Three genes overlapped and were dysregulated in both individuals diagnosed with ASD and in asdMO. An ASD-associated gene expression pattern was often observed in both asdMO and individuals with ASD even though they had no symptoms above clinical threshold of ASD (expression of the 19 and 57 genes was changed in a parallel direction; Kuwano et al., 2011).

Glatt et al. identified 60 infants and toddlers at risk for ASDs, 34 at risk for language delay, 17 at risk for global developmental delay, and 68 typically developing children. One hundred and fifty four probes showed significant dysregulation in ASD, and a log 2-fold change. The most accurate support vector machine utilized the magnitude of the expression of 48 probes to classify 71% of ASD and control subjects across 10 subsets of discovery sample into their appropriate diagnostic categories. Of 30 individuals diagnosed with ASD, 27 were correctly classified by this support vector machine as having ASD, 23/34 control subjects were correctly classified as controls. The list of 48 probes making up the best support vector machine classifier of ASDs was most significantly enhanced with genes related to immune responses, genes of the hemoglobin complex, and genes with guanine- or guanylate-binding affinity (Glatt et al., 2012).

Kong et al. performed a genome-wide expression profile of the blood from 20 proband-unaffected sibling pairs, and 18 unrelated controls. One hundred and eighty nine probe sets that represented 163 unique genes (including 2 previously reported ASD candidate genes) were significantly changed between probands and siblings—84 probands were up-regulated compared to unaffected siblings (Kong et al., 2013).

Segura et al. looked at neurotrophin blood-based gene expression and social cognition analysis by obtaining whole blood from 21 adults and adolescents diagnosed as ASD, as well as from 10 controls. Social cognition abilities of subjects with ASD and controls were determined according to three Theory of Mind tests (RME, Faux pas test, The Happé stories). They found that NT3 and NT4 mRNA expression in the whole blood was significantly lower in ASD patients compared to healthy controls (*P* < 0.05). They also found that P75NTR mRNA expression was significantly higher in ASD patients than in controls. The ASD group received lower scores in three Theory of Mind tasks compared to the control group, which indicates that social cognition impairments in association with the ASD phenotype, yet no correlations were observed between neurotrophins and their receptors expressions and measures of Theory of Mind (Segura et al., 2015).

## GI and ASD background

Gastrointestinal (GI) symptoms are common in children with ASD compared with typically developing children and those with other developmental delays. Some controversial studies suggest that as many as 70% of children with ASD exhibit chronic GI-related symptoms (Walker et al., 2013).

## Gastrointestinal tissue gene expression studies

Williams et al. looked at impaired carbohydrate digestion and transport and mucosal dysbiosis in the intestines of children diagnosed with ASD and gastrointestinal disorders. They found that the levels of three brush border disaccharidases (sucrase isomaltase [SI], maltase glucoamylase [MGAM], and lactase [LCT]) were all significantly decreased in children with ASD and GI complaints (ASD-GI). Within the ASD-GI group, 86.7, 80, and 80% of children had lower transcript levels in SI, MGAM, and LCT respectively. Almost all (14/15, or 93.3%) ASD-GI children had deficiencies in at least one disaccharidase enzyme; 80% had deficiencies in 2 or more enzymes; and 73.3% had deficiencies in all three enzymes. Real-time polymerase chain reaction (RT-PCR) revealed a significant decrease in 2 hexose transporters: ileal SGLT1 mRNA and GLUT2 mRNA in ASD-GI children. For SGLT1, 73.3% of ASD-GI children had deficient transcript levels, and 73.3% of ASD-GI children had deficient GLUT2 transcript levels, relative to control-GI children. In total, 93.3% (14/15) of ASD-GI children had mRNA deficiencies in at least one of the five genes involved in carbohydrate digestion or transport; 66.7% (10/15) had mRNA deficiencies in all five genes (Williams et al., 2011).

Similarly, Walker et al. looked at subjects including children with ASD and three typically developing groups including (1) children who underwent diagnostic ileocolonoscopy for chronic GI symptoms in which no histopathology; (2) children with Crohn's disease (3) and children with ulcerative colitis. Pairwise analysis between the ileal mucosa from ASD-GI and non-inflamed control samples resulted in 1409 differentially expressed transcripts unique to the ASD-GI samples. Pairwise analysis between inflamed colonic mucosa from ASD-GI children and non-inflamed control samples resulted in 1189 differentially expressed transcripts unique to ASD-GI samples. The overlap between the 2 sets (ileum and colon) resulted in 178 transcripts that were exclusively differentially expressed in both ileal and colonic tissues form the ASDGI population. When these 178 transcripts were analyzed using Ingenuity Pathway Analysis software, three of the top associated biological functions were inflammatory disease, endocrine system development and function, and digestive system development and function (Walker et al., 2013).

## Adult olfactory stem cell gene expression studies

Féron et al. used adult nasal olfactory stem cells from nine adults with severe ASD and low developmental disabilities (DSM-5), plus two adults with mild ASD and no or mild cognitive abilities (Asperger syndrome or high functioning ASD) paired with 11 age and gender matched controls. Gene microarray analysis highlighted 156 genes that were differentially expressed in at least one ASD patient, of which 31 were dysregulated in more than 33% of the cohort (9 out of the 156 genes have been previously associated with ASD). They found that MOCOS, an enzyme involved in purine metabolism, is downregulated in most ASD individuals (8/11), compared to controls (Féron et al., 2016).

## Hair follicle gene expression studies

Maekawa et al. utilized scalp hair follicles as a source of biomarker genes and found that the gene CNTNAP2 showed significantly decreased expression in samples from subjects with ASD compared with control follicles (Maekawa et al., 2015).

## Gene expression analysis: comorbidities

A few of the gene expression studies looked at people diagnosed with ASD and comorbidities.

### ASD and Fragile X/Dup 15q

Nishimura et al. performed genome-wide expression profiling of LCL in order to distinguish different forms of ASD and to reveal shared pathways. Individuals with ASD both with and without FMR1-FM or dup (15q) were compared to TD male controls. The combination of ANOVA, SAM and RankProd, isolated 120 genes in ASD with FMR1-FM, and 80 genes in ASD dup (15q), 68 genes were found to be dysregulated in both ASD with FMR1-FM and dup (15q) (so 52 genes were selectively dysregulated only in ASD with FMR1-FM, and 12 genes were selectively dysregulated only in ASD with dup (15q). They also found a potential molecular connection between FMR1-FM and dup (15q), the cytoplasmic FMR1 interaction protein 1 (CYFIP1), which was up-regulated in dup (15q) patients. Expression of JAKMIP1 and GPR155 was significantly dysregulated in the 27 males with ASD when compared with their siblings without ASD. JAKMIP1 is a gene known to be related to microtubule transport. Genes related to chaperone and protein folding were enriched in the 52 genes selectively dysregulated in ASD with FMR1-FM; genes related to RNA binding and mRNA metabolism were also enriched in this set (this is consistent with FMRP protein's function as an RNA binding protein important in regulatory translation) (Nishimura et al., 2007).

### ASD and ADHD

Taurines et al. looked at altered mRNA expression of monoaminergic specific genes in the blood of children with ADHD and ASD. They found a significant group difference with decreased DRD5- level in ASD patients when compared with controls and to patients diagnosed with ADHD. *Post-hoc* analyses demonstrated reduced DRD4-levels in the group of both ADHD patients and ASD patients when compared with healthy controls (Taurines et al., 2011).

## Gene expression in ASD-summary (Table 2)

### Sample source types

When the gene expression studies are viewed together independent of source type, over 100 genes are found in more than one study. Interestingly, whether the gene is up-regulated or down-regulated is independent of the source of the gene sampled and can even vary within the same source type in different studies. For example, the gene NDUFB5 was found to be upregulated in LCL derived cell line RNA's (Talebizadeh et al., 2014), but downregulated in three different regions of post-mortem brain tissue (Anitha et al., 2012). The gene NEURL3 was up-regulated in peripheral blood in a study conducted by Chien et al. (2013), but down-regulated in peripheral blood in a study conducted by Kong et al. (2013). However, most genes that were up or down-regulated followed the same pattern in different studies across sample source types.

**Table 2:** **Gene expression changes in ASD detected in multiple independent studies in various tissues**.

**Gene symbol**	**Blood/LCL study**	**Brain study**	**Intestinal biopsy study**	**Expression change in ASD**
ABHD3	Kong et al., 2013	Garbett et al., 2008		Upregulated
ACTG2		Ziats and Rennert, 2013	Walker et al., 2013	Downregulated
ADM		Garbett et al., 2008; Voineagu and Eapen, 2013		Upregulated
AHI1		Garbett et al., 2008; Voineagu and Eapen, 2013		Downregulated
ALAD		Chow et al., 2012	Walker et al., 2013	Downregulated
ALPK1	Nishimura et al., 2007; Talebizadeh et al., 2014			Upregulated
ANKRD22	Glatt et al., 2012; Ivanov et al., 2015			Upregulated in Ivanov et al. Downregulated in Glatt et al.
ANXA1	Chien et al., 2013	Garbett et al., 2008; Voineagu and Eapen, 2013; Ziats and Rennert, 2013		Upregulated in Garbett et al. and Ziats et al. Downregulated in Chien et al.
AQP4		Garbett et al., 2008; Voineagu and Eapen, 2013		Upregulated
ATF3	Hu et al., 2006		Walker et al., 2013	Upregulated in Hu et al. Downregulated in Walker et al.
BAG3		Garbett et al., 2008; Voineagu and Eapen, 2013		Upregulated
C20orf7		Chow et al., 2012; Ginsberg et al., 2012		Downregulated
C5orf16		Garbett et al., 2008; Voineagu and Eapen, 2013		Downregulated
CCL17	Nishimura et al., 2007		Walker et al., 2013	Upregulated
CD160	Gregg et al., 2008; Enstrom et al., 2009			Upregulated
CHI3L1	Chien et al., 2013	Garbett et al., 2008		Upregulated in Garbett et al. Downregulated in Chien et al.
CLIC1		Garbett et al., 2008; Voineagu and Eapen, 2013		Upregulated
CMKOR1	Nishimura et al., 2007	Garbett et al., 2008; Voineagu and Eapen, 2013		Upregulated
CNN3		Garbett et al., 2008; Voineagu and Eapen, 2013		Upregulated
COL4A1		Garbett et al., 2008; Voineagu and Eapen, 2013		Upregulated
COX7B		Ginsberg et al., 2012; Anitha et al., 2012		Downregulated
CSDA		Garbett et al., 2008; Voineagu and Eapen, 2013		Upregulated
CTNNB1	Kong et al., 2013	Chow et al., 2012		Upregulated in Kong et al. Downregulated in Chow et al.
CX3CR1	Gregg et al., 2008; Enstrom et al., 2009	Ziats and Rennert, 2013		Upregulated
CXCL10	Chien et al., 2013	Chow et al., 2012		Upregulated
CXCR4	Chien et al., 2013	Chow et al., 2012		Upregulated
CYC1		Ginsberg et al., 2012; Anitha et al., 2012		Downregulated
CYFIP1	Nishimura et al., 2007; Talebizadeh et al., 2014			Upregulated
DLX1		Garbett et al., 2008; Voineagu and Eapen, 2013		Downregulated
DNASE1L3	Chien et al., 2013	Chow et al., 2012		Downregulated
DRD4	Emanuele et al., 2010; Taurines et al., 2011			Upregulated in Emanuele et al. Downregulated in Taurines et al.
FAM46C	Nishimura et al., 2007; Chien et al., 2013			Upregulated in Chien et al. Downregulated in Nishimura et al.
FOSL1	Ivanov et al., 2015	Chow et al., 2012		Upregulated in Chow et al. Downregulated in Ivanov et al.
GAD1	Chien et al., 2013	Zhubi et al., 2014		Downregulated
GADD45B		Garbett et al., 2008; Voineagu and Eapen, 2013		Upregulated
GPR56	Gregg et al., 2008	Ginsberg et al., 2012		Upregulated
GRIA3	Chien et al., 2013	Chow et al., 2012		Downregulated
GZMB	Gregg et al., 2008; Enstrom et al., 2009; Chien et al., 2013			Upregulated
HCK	Hu et al., 2006; Chien et al., 2013; Talebizadeh et al., 2014			Upregulated in Talebizadeh et al., Downregulated in Hu et al. and Chien et al.
HIST1H1C		Garbett et al., 2008; Voineagu and Eapen, 2013		Upregulated
HIST1H2BD		Garbett et al., 2008; Voineagu and Eapen, 2013		Upregulated
HIST1H3H	Nishimura et al., 2007	Chow et al., 2012		Upregulated in Chow et al. Downregulated in Nishimura et al.
HLA-DQA1	Gregg et al., 2008; Stamova et al., 2011			Downregulated
HSPB1		Garbett et al., 2008; Ginsberg et al., 2012		Upregulated
IFITM2		Garbett et al., 2008; Voineagu and Eapen, 2013		Upregulated
IFITM3		Garbett et al., 2008; Voineagu and Eapen, 2013		Upregulated
IGF2BP1	Ivanov et al., 2015		Walker et al., 2013	Upregulated in Walker et al. Downregulated in Ivanov et al.
IGHA1	Gregg et al., 2008; Chien et al., 2013			Upregulated
IGHG1	Hu et al., 2006; Gregg et al., 2008; Chien et al., 2013			Upregulated in Chien et al. and Gregg et al. Downregulated in Hu et al.
IL2RA	Chien et al., 2013		Walker et al., 2013	Upregulated
IL2RB	Gregg et al., 2008; Enstrom et al., 2009			Upregulated
ITGB2	Gregg et al., 2008; Enstrom et al., 2009			Upregulated
KIF1B	Hu et al., 2006; Talebizadeh et al., 2014	Garbett et al., 2008		Upregulated in Garbett et al. and Talebizadeh et al. Downregulated in Hu et al.
KIR3DL2	Gregg et al., 2008; Enstrom et al., 2009			Upregulated
KSP37	Gregg et al., 2008; Enstrom et al., 2009			Upregulated
LAMP2		Chow et al., 2012	Walker et al., 2013	Downregulated
LRP6	Chien et al., 2013; Talebizadeh et al., 2014			Downregulated
MeCP2	Kuwano et al., 2011	James et al., 2014; Zhubi et al., 2014^**^		Upregulated in Kuwano et al. and Zhubi et al. Downregulated in James et al.
MIA	Nishimura et al., 2007		Walker et al., 2013	Upregulated in Nishimura et al. Downregulated in Walker et al. (Colon)
MKNK2		Garbett et al., 2008; Voineagu and Eapen, 2013		Upregulated
MSI2		Garbett et al., 2008; Voineagu and Eapen, 2013		Upregulated
MSN		Garbett et al., 2008; Voineagu and Eapen, 2013		Upregulated
NDUFA2		Ginsberg et al., 2012; Anitha et al., 2012		Downregulated
NDUFB3		Ginsberg et al., 2012; Anitha et al., 2012		Downregulated
NDUFB5	Talebizadeh et al., 2014	Anitha et al., 2012		Upregulated in Talebizadeh et al., Downregulated in Anitha et al.
NEURL3	Kong et al., 2013; Chien et al., 2013			Upregulated in Chien et al. Downregulated in Kong et al.
NKG7	Gregg et al., 2008; Enstrom et al., 2009			Upregulated
NP		Garbett et al., 2008; Voineagu and Eapen, 2013		Upregulated
P2RX5	Hu et al., 2006; Chien et al., 2013			Upregulated
P4HA1		Garbett et al., 2008; Voineagu and Eapen, 2013		Upregulated
PALLD		Garbett et al., 2008; Voineagu and Eapen, 2013		Upregulated
PAM	Gregg et al., 2008; Enstrom et al., 2009			Upregulated
PARP9	Glatt et al., 2012	Garbett et al., 2008		Upregulated in Garbett et al. Downregulated in Glatt et al.
PIR		Garbett et al., 2008; Voineagu and Eapen, 2013		Upregulated
PITPNC1	Hu et al., 2006; Nishimura et al., 2007	Garbett et al., 2008; Voineagu and Eapen, 2013		Upregulated in Nishimura et al. Garbett et al. and Voineagu et al. Downregulated in Hu et al.
PLEKHC1		Garbett et al., 2008; Voineagu and Eapen, 2013		Upregulated
PRF1	Gregg et al., 2008; Enstrom et al., 2009			Upregulated
PTGDR	Gregg et al., 2008; Enstrom et al., 2009			Upregulated
PTTG1IP		Garbett et al., 2008; Voineagu and Eapen, 2013		Upregulated
PXDN	Stamova et al., 2011; Chien et al., 2013			Upregulated in Chien et al. Downregulated in Stamova et al.
RELN		Chow et al., 2012; Khan et al., 2014; Zhubi et al., 2014		Upregulated in Khan et al. Downregulated in Chow et al. and Zhubi et al.
RPS21		Garbett et al., 2008; Ginsberg et al., 2012		Upregulated in Garbett et al. Downregulated in Ginsberg et al.
S100A10		Garbett et al., 2008; Voineagu and Eapen, 2013		Upregulated
SCARA3		Garbett et al., 2008; Voineagu and Eapen, 2013		Upregulated
SDC2		Garbett et al., 2008; Ziats and Rennert, 2013		Upregulated
SERPINA1	Chien et al., 2013	Chow et al., 2012		Upregulated in Chow et al. Downregulated in Chien et al.
SERPINH1		Garbett et al., 2008; Chow et al., 2012; Ziats and Rennert, 2013		Upregulated
SERTAD1		Garbett et al., 2008; Voineagu and Eapen, 2013		Upregulated
SFTPA2		Chow et al., 2012	Walker et al., 2013	Upregulated in Chow et al. Downregulated in Walker et al.
SH2DIB/EAT2	Gregg et al., 2008; Enstrom et al., 2009			Upregulated
SHANK3		Yasuda et al., 2011; Chana et al., 2015		Downregulated
SLC38A2	Hu et al., 2006; Kong et al., 2013			Upregulated
SLC9A9	Talebizadeh et al., 2014	Garbett et al., 2008; Voineagu and Eapen, 2013		Upregulated
SPON2	Gregg et al., 2008; Enstrom et al., 2009			Upregulated
STOM	Glatt et al., 2012	Garbett et al., 2008; Ziats and Rennert, 2013		Upregulated in Garbett et al. Downregulated in Glatt et al. and Ziats et al.
SYCE1	Kong et al., 2013	Chow et al., 2012		Downregulated
TAGLN2		Garbett et al., 2008; Voineagu and Eapen, 2013		Upregulated
TAP1	Glatt et al., 2012	Garbett et al., 2008; Voineagu and Eapen, 2013		Upregulated in Garbett et al. Downregulated in Glatt et al.
TBX21	Gregg et al., 2008; Enstrom et al., 2009			Upregulated
TET1		James et al., 2014; Zhubi et al., 2014		Upregulated
TIMP1		Garbett et al., 2008; Voineagu and Eapen, 2013		Upregulated
TMBIM1		Garbett et al., 2008; Voineagu and Eapen, 2013		Upregulated
TMEM40	Kong et al., 2013; Ivanov et al., 2015			Downregulated
TNFRSF19	Chien et al., 2013	Chow et al., 2012		Downregulated
TNPO1		Garbett et al., 2008; Voineagu and Eapen, 2013		Upregulated
UBD	Chien et al., 2013		Walker et al., 2013	Upregulated
WWTR1	Chien et al., 2013	Garbett et al., 2008		Upregulated in Garbett et al., Downregulated in Chien et al.
YAP1		Garbett et al., 2008; Voineagu and Eapen, 2013		Upregulated
ZFP36L1		Garbett et al., 2008; Voineagu and Eapen, 2013		Upregulated

** *James et al. (2014). looked at MeCP2 binding to the EN-2 promoter, while Zhubi et al. (2014) looked at MeCP2 binding to the GAD1 & RELN promoters*.

### Pathways (Table 3)

One of the aims of gene expression studies is to look for multiple genes in the same pathway. This should provide some clues to the underlying mechanism of the disease. From the studies surveyed, five pathways were found three times across different studies, and eight pathways were found twice. The pathways that were found three times included: cell cycle, cell death, GI disease, immune function, and neurogenesis. All of these areas are already areas of extensive research in ASD and therefore, they are not-surprising. The pathways that were found twice were: alternative splicing, arrhythmogenic right ventricular cardiomyopathy, cellular assembly and organization, cell-to-cell signaling and interaction, gap junction, inflammation, small molecule biochemistry, and ubiquitin mediated proteolysis. While inflammation and cell signaling are already areas of research for ASD, the potential relationship between the other pathways and ASD deserves further consideration. A more recent paper by Ivanov et al. (2015) highlights a number of other pathways and their importance in understanding the function of genes including the Wnt pathway and the calcium pathway, which is involved in the development of the nervous system and deserves further investigation regarding its potential role in ASD. Similarly, Wen et al. (2016) found the calcium signaling pathway to be a very active pathway in ASD. The precise role of calcium signaling in ASD and its potential relationship to other common metabolic disturbances in ASD demands further research.

**Table 3:** **List of main pathways affected**.

**Paper**	**Software**	**List of main pathways affected**
Alter et al., 2011	DAVID analysis	Alternative splicing, splice variant, zinc-finger, phosphoprotein, zinc, metal-binding, zinc-ion binding, dna-binding, ubiquitin mediated proteolysis, nucleus, transcription, transition metal ion binding, chromosomal rearrangement, ubl conjugation pathway, transcription regulation, coiled coil, regulation transcription, compositionally biased region: Ser-rich
Chien et al., 2013	DAVID analysis	Long-term depression, Cytokine-cytokine receptor interaction, Vascular smooth muscle contraction, Arrhythmogenic right ventricular cardiomyopathy (ARVC), glycerophospholipid metabolism, allograft rejection, Jak-STAT signaling pathway, Hematopoietic cell lineage, Gap junction, T cell receptor signaling pathway, RIG-I-like receptor signaling pathways, Ubiquitin mediated proteolysis, Intestinal immune network for IgA production, Type II diabetes mellitus, Leukocyte transendothelial migration, GnRH signaling pathway
	EMA (Easy Microarray data Analysis)	Long-term depression, Amoebiasis, Vascular smooth muscle contraction, Cytokine-cytokine receptor interaction, Arrhythmogenic right ventricular cardiomyopathy (ARVC), Intestinal immune network for IgA production, Endocytosis, Aldosterone-regulated sodium reabsorption, African trypanosomiasis, Graft-vs.-host disease
	GSEA Gene Set Enrichment Analysis	Arrhythmogenic right ventricular cardiomyopathy ARVC, terpenoid backbone biosynthesis, glycerophospholipid metabolism, vascular smooth muscle contraction, alpha linolenic acid metabolism, tight junction, inositol phosphate metabolism, cytosolic dna sensing pathway, renal cell carcinoma, cell receptor signaling pathway, chronic myeloid leukemia
Chow et al., 2012		
Young autistic/control	MetaCore software suite	DNA damage-response, cell cycle and apoptosis-related pathways
	DAVID analysis	DNA damage/cell cycle, apoptosis, and immune signaling and neurogenesis and neural development
Adult autistic/control	MetaCore software suite	Cell differentiation, mitogenic signaling and apoptosis genes
All autistic and control cases independent of age		DNA-damage response, apoptosis and immune system response functions
Enstrom et al., 2009	DAVID analysis	NK function, cellular proliferation, and leukocyte function
Féron et al., 2016	IPA analysis	Developmental disorders, gastrointestinal diseases, purine metabolism, inflammation
Garbett et al., 2008	GSEA Gene Set Enrichment Analysis	Antigen-specific immune response, inflammation, cell death, autoimmune diseases, migration, and targeting of the immune response to specific cells
Ginsberg et al., 2012	DAVID analysis	Mitochondrial oxidative phosphorylation, protein translation, synapse/neurotransmitters, vesicle transport, brain patterning,
	IPA analysis	Oxidative phosphorylation
Glatt et al., 2012	DAVID analysis	Genes related to immune response, genes of the hemoglobin complex, and genes with guanine- or guanylate-binding affinity
Gregg et al., 2008	DAVID analysis	Natural killer cell-mediated cytotoxicity
	IPA analysis	Natural killer cell signaling, IL-2 signaling, serotonin receptor and dopamine receptor signaling, retinol and methionine metabolism
Hu et al., 2006	IPA analysis	Neurological development and function, neuronal signaling, extension of neurites, myelination, VEGF-induced release of nitric oxide, neurogenesis, survival of Purkinje cells, apoptosis of neurons, development of septum, TNF and other cytokines
Hu et al., 2009	IPA analysis	Synaptic transmission, neurogenesis, neurulation, long-term potentiation (learning), protein ubiquitination, brain function, molecular and cellular functions, cell death, small molecule biochemistry, free radical scavenging, cellular function and maintenance, liver toxicity, circadian rhythm, and androgen sensitivity
Ivanov et al., 2015	KEGG pathway analysis	Calcium signaling pathway, Amphetamine addiction, Leishmaniasis, GABAergic synapse, Retrograde endocannabinoid signaling, MAPK signaling pathway, Arrhythmogenic right ventricular cardiomyopathy (ARVC), Cholinergic synapse, GnRH signaling pathway, Glutamatergic synapse, Serotonergic synapse, Dopaminergic synapse, Steroid biosynthesis, Steroid biosynthesis, Wnt signaling pathway, Leukocyte transendothelial migration, Hypertrophic cardiomyopathy (HCM), Dilated cardiomyopathy, Asthma, Influenza A, Carbohydrate digestion and absorption and Endocrine and other factor-regulated calcium reabsorption
Kong et al., 2013	GSEA Gene Set Enrichment Analysis	Ribosome and spliceosome pathways, neuroactive ligand receptor interaction pathway, calcium signaling pathway, and Gap junction
Kuwano et al., 2011		
ASD/control group	IPA analysis	Cell morphology, cellular assembly and organization, nerve system development and function
asdMO/asdMO	IPA analysis	Cancer, RNA post-transcriptional modification, reproductive system disease, protein synthesis, immune functions
Mahfouz et al., 2015	DAVID analysis	Synaptogenesis, regulation of apoptosis, regulation of cell death, GABAergic neurons, neuron projection, neuron differentiation, cell morphogenesis, learning/memory, behavior, mental retardation, epilepsy, mitochondrial function, protein translation, ubiquitination, synapse formation and elimination, protein turnover, ion channel, neurotransmitter receptor activity, and mitochondrial function
Nishimura et al., 2007	DAVID analysis	Cell communication and signal transduction, immune response, defense response
	IPA analysis	Cell cycle, cellular movement, and cell-to-cell signaling and interaction
Pramparo et al., 2015	Metacore GeneGo analysis	Apoptosis/Apoptotic nucleus, Immune response/Antigen presentation, Immune response/Phagocytosis, Immune Response/TCR signaling, Translation/Translation initiation, Inflammation/Interferon signaling, Apoptosis/Anti-Apoptosis via NF-kb, Cell Adhesion/Leukocyte chemotaxis, Inflammation/IFN-gamma signaling
Stamova et al., 2011	IPA analysis	Cellular Assembly and organization, cellular compromise, small molecule biochemistry, vitamin and mineral metabolism, cell death, neuronal development, neuronal survival
Talebizadeh et al., 2014	DAVID analysis	Protein-lipid modification
Tian et al., 2011	IPA analysis	Immunological and inflammatory disease processes, cell-cell signaling, antigen presentation, cell cycle, development and growth, proliferation, mitochondrial dysfunction pathways
Voineagu and Eapen, 2013	GO gene ontology enrichment analysis	Synaptic function, immune and inflammatory function
Walker et al., 2013		
Ileal Mucosa	PCA (Principal component analysis)	Gastrointestinal disease, inflammatory response, humoral immune response, tissue morphology, digestive system development and function, O-Glycan Biosynthesis, Propanoate Metabolism, Arginine and Proline Metabolism, and Alanine and Aspartate Metabolism
Colonic Mucosa	PCA (Principal component analysis)	Gastrointestinal disease, neurological disease, behavior, organ development, Atherosclerosis Signaling, Factors Promoting Cardiogenesis in Vertebrates and Mitotic Roles of Polo-Like Kinase
	IPA analysis	Inflammatory disease, endocrine system development and function and digestive system development and function, Granzyme A Signaling, Athersclerosis Signaling, Valine, Leucine and Isoleucine Degradation and Clathrin-mediated Endocytosis Signaling
Wen et al., 2016	KEGG Pathway Analysis	MAPK signaling pathway, Calcium signaling pathway, Cell signaling, cell structure/transport, metabolism, neural, immune, cancer, cardiac disease, metabolic disease, Cell Adhesion Molecules, Wnt signaling pathway, mTOR signaling pathway, Focal adhesion, Regulation of actin cytoskeleton, Ubiquitin mediated proteolysis, Long-term potentiation, Axon guidance, and neurodegenerative diseases
Ziats and Rennert, 2013	DAVID analysis	Extracellular matrix formation/glycoproteins, immune response, chromatin, and cell cytoskeleton

### Specific genes/pathways already implicated in ASD or in processes relevant to ASD

There are numerous examples of specific genes that have been shown to be altered between ASD and control samples. All have been shown to be directly related to important processes that when reduced or altered, can be connected to ASD. These genes can be roughly subdivided into genes related to the brain and genes related to the immune system.

### Brain related genes

Hu et al. found the protein ASS to be upregulated in the autism samples. ASS controls the rate-limiting step involved in nitric oxide (NO) production via regeneration of arginine from citrulline, a by-product of the nitric oxide synthetase (NOS) reaction. Since NO is a important signaling molecule in the brain and has been implicated in several disorders, including ASD, thus the increased expression of ASS may be potentially relevant to the ASD phenotype (Hu et al., 2006).

Based on the findings of Nishimura et al., since CYFIP1, which was shown to be upregulated in dup (15q) patients is known to counteract FMRP, they reason that the induction of CYFIP1 in dup (15q) might elucidate some of the significant overlap between ASD with FMR1-FM and with dup (15q). They also found that JAKMIP1 was significantly induced in ASD with FMR1-FM and had a positive trend in ASD with dup (15q), suggesting that JAKMIP1 could represent a commonly dysregulated pathway. This gene is a particularly biologically important candidate, given its putative role in GABAB receptor expression and microtubule networks (Nishimura et al., 2007).

Since Taurines et al. found reduced expression in ASD probands of DRD5 which is expressed in the hippocampus associated areas and is thought to be important in the induction of long term potentiation related to novel events, it can be suggested that the reduced expression could give insight into the fact that probands have less expression of an important hippocampal gene (Taurines et al., 2011).

Kuwano et al. found that mRNA levels of ITGA2B encoding glycoprotein (GP) αIIβ were upregulated both in individuals with ASD and in asdMO; GPαIIβ forms αIIbβ3 integrin with ITGB3, an ASD-susceptible gene. Since αIIbβ3 integrin has an critical role in cell morphology, including synapse maturation, the increased expression of ITGA2B mRNA might change cellular morphology of peripheral cells in mothers having children with ASD as well as subjects with ASD (Kuwano et al., 2011).

Chow et al. showed that the A2A receptor-signaling pathway was the top dysregulated pathway in the young autistic brain. Adenosine receptors are crucial for both brain development and function including the regulation of neuronal stem cell proliferation, synaptic plasticity, motor function, cognition and emotion-related behaviors (Chow et al., 2012).

Voineagu et al. identified that A2BP1/FOX1, a neural and muscle specific alternative splicing regulator (and the only splicing factor previously implicated in ASD) was down-regulated in several individuals with ASD (Voineagu and Eapen, 2013).

The reports of James et al. showed that elevated 5-hmC in the EN-2 promoter is correlated with a significant decrease in repressive MeCP2 and histone H3K27me3 which appear to override 5-mC hypermethylation. These epigenetic changes are thought to loosen enhancer region chromatin which would facilitate enhancer binding and promote sustained upregulation of EN-2 expression. Since perinatal EN-2 downregulation is crucial for normal Purkinje cell differentiation and cerebellar patterning, the consistent postnatal overexpression of EN-2 suggests that the shutting of this programmed developmental window may have been missed in some individuals with ASD because of epigenetic abnormalities (James et al., 2014).

NT3&NT4, which were down to have lower expression in ASD patients compared to controls by Segura et al. play a crucial role in the development of the climbing fiber system of the cerebellar Purkinje cells (PCs), and NT3 selectively increased their survival. PC's are the primary efferent neurons of the cerebellar cortex, and its potential involvement in ASD has long been proposed. Neuropathological studies have shown significant reductions in the number and size of PCs in ASD post-mortem brain. Therefore, they hypothesize that reduced NT3 levels in the periphery in ASD patients might reflect altered expression in the CNS which may be associated with a loss of PC that result in altered cerebellar cortical efferent signals (Segura et al., 2015).

CNTNAP2, which encodes the contactin associated protein-like 2, which was found have significantly decreased gene expression in Maekawa et al. is one of the most intense ASD susceptibility genes with supporting evidence from several independent studies (Peñagarikano and Geschwind, 2012). CNTNAP2, a neurexin family protein that acts as a neuronal adhesion molecule and receptor. It was found to be a direct neural target of the human FOXP2 protein, and mutations of FOXP2 and CNTNAP2 were linked to language and speech disorders in ASD (Maekawa et al., 2015). The FOXP1 gene, which was found to be elevated in ASD subjects according to Chien et al. functions as a transcription repressor, forms a heterodimer with FOXP2, and is co-expressed with FOXP2 in numerous brain regions, suggesting close functional cooperation between the two proteins. FOXP1 is extensively expressed in the developing and mature brain and has been suggested to be important for brain development and function. Based on their data, it indicates that associations among FOXP1, FOXP2, and CNTNAP2 genes may play an important role underlying the pathogenesis of syndromic and non-syndromic ASD. They inferred that increased FOXP1 gene expression may lead to increased FOXP2 gene expression through a feedback mechanism, which may in turn reduce the gene expression of CNTNAP2 in patients with ASD (Chien et al., 2013).

Feron et al. found MOCOS to be downregulated in most ASD individuals as compared to controls. *In vivo* and *in vitro* engineered models indicate that altered expression of MOCOS results in neurotransmission and synaptic defects. MOCOS expression also induces increased oxidative stress sensitivity. Metabolic disorders of purine metabolism have been shown to affect the nervous system and are able to induce autistic features (Féron et al., 2016).

### Immune related genes

Gregg et al. found that SH2D1B/EAT2, one of the 11 differentially expressed genes they found to overlap in all 3 groups (AU vs. GP, A-E vs. GP, and A-R vs. GP), is mostly expressed in natural killer cells as well as macrophages, B cells, and dendritic cells, and has been theorized to suppress natural killer cell activity through the binding of protein tyrosine phosphatases, inhibitory kinases, or ubiquitin ligases. Abnormalities in RUNX3 (one of the 55 differentially expressed genes in AU vs. GP) function in leukocytes and is associated with sudden development of colitis and gastric mucosal hyperplasia and might be relevant to ASD since a small group of children with ASD appear to have gastrointestinal abnormalities (Gregg et al., 2008).

The findings of Enstrom et al. suggest possible dysfunction of natural killer cells in children with ASD, and the data suggests that circulating natural killer cells in ASD are persistently activated rather than quiescent (Enstrom et al., 2009).

Reduced expression of NLGN3 and SHANK3 genes in lymphoblasts of individuals with ASD is consistent with previous reports indicating that mutations of these genes cause reduced expression or loss of function of the protein. Yasuda et al. found that both these genes were found to be decreased in individuals in ASD (Yasuda et al., 2011).

One of the 48 biomarkers in the optimized support vector machine classifier by Glatt et al. IF116, was previously found to be dysregulated in the postmortem temporal cortex of subjects with ASD (Garbett et al., 2008; Glatt et al., 2012).

## General limitations in RNA-gene expression studies: a critical appraisal of the data

### Variance

One limitation of mRNA gene expression studies is variance. While gene specific approaches are helpful, they may ignore changes known as variance occurring at the global level of gene expression regulation. Global levels of gene expression regulation are crucial for understanding the underlying basis of diseases such as ASD where multiple systems are affected. For example, the associated increased risk of ASD in children of older fathers could be mediated by changes in global levels of gene expression regulation, or by paternally transmitted age related factors that are linked to changes in the global regulation of gene expression (Alter et al., 2011). Thus, it is possible that a common mediator, a change at the global level of gene expression regulation, could offer an all-encompassing explanation for multi-systemic effects of the disease.

### Tissue source

According to Chien et al. there are several disadvantages with the use of post-mortem brain tissue in gene expression studies (Chien et al., 2013). They point out that using fresh brain tissue from living patients is not always practical, and as a surrogate for brain tissue, several studies have instead utilized peripheral blood cells and LCL. They also state that there is a moderate correlation of gene expression has been reported between peripheral blood cells and brain tissue in humans which supports the usefulness of peripheral blood cells instead of brain-tissue for gene expression studies. Yet Mahfouz et al. (2015), argue that due to the nature of the pathology of ASD, which affects brain regions and the connection between various brain regions, there are advantages to post-mortem gene studies from brain tissue over peripheral blood studies. Kuwano et al. concluded that gene expression profiling of LCL are well documented because of their homogeneity (Kuwano et al., 2011).

Segura et al. explained that their rationale to use blood as opposed to post-mortem brain tissue was due to the limited access of tissue from the central nervous system (CNS) in humans (Segura et al., 2015). They also brought reports of a potential correlation between neurotrophin expression in CNS and the periphery, which would suggest that taking blood in order to study neurotrophins would be similarly effective to using brain tissue. Similarly, Pramparo et al. (2015) emphasized the advantages of peripheral blood, and were successful in identifying 2765 genes from a peripheral blood source from a variety of pathways including apoptosis, the immune response, and genes involved in translation.

According to Hu et al. while studies that used brain tissue to better understand the mechanistic basis for ASD could be informative; this method of study is not an appropriate target for diagnostic assays. They counter that diagnostic assays should ideally be taken from easily obtainable samples such as the patient's blood (Hu et al., 2006). However, Talebizadeh et al. acknowledge that LCL samples may not be ideal to use in order to investigate brain-related genes, but they may still be helpful for understanding at least a subset of brain-related changes (Talebizadeh et al., 2014).

## Limitations specific to ASD gene expression studies: a critical appraisal of the data

The clinical heterogeneity of ASD presents a challenge any time studies attempt to find patterns across the spectrum. Complicating matters even more, the ASD gene expression studies relied on a variety of diagnostic methods to define ASD (Tables 4A,B). The lack of consistency between the diagnostic criteria and the subjectivity of the behavioral methods of diagnosis limit the ability to extrapolate the data to the broader ASD population.

**Table 4A:** **Diagnostic tests part 1**.

**Paper/diagnostic test**	**DSM-4**	**DSM-5**	**ADOS**	**ADI-R**	**CARS**	**IQ scores**	**SCQ**	**CSBS DP**	**Mullen scales of early learning**	**Bayley scale of infant and toddler development**	**Vineland adaptive behavior scales**	**ToM social cognition test**	**Peabody Picture Vocabulary Test (PPVT)**
Alter et al., 2011	✓		✓	✓									
Chien et al., 2013	✓			✓									
Chow et al., 2012			✓	✓									
Emanuele et al., 2010	✓				✓								
Enstrom et al., 2009	✓		✓	✓			✓						
Féron et al., 2016		✓									✓		
Ginsberg et al., 2012	✓		✓	✓		✓							
Glatt et al., 2012			✓					✓	✓				
Gregg et al., 2008	✓		✓	✓									
Hu et al., 2006			✓	✓									✓
Hu et al., 2009				✓									
Ivanov et al., 2015				✓	✓								
James et al., 2014	✓			✓									
Kong et al., 2013			✓	✓									
Kuwano et al., 2011	✓					✓							
Maekawa et al., 2015	✓			✓									
Nishimura et al., 2007			✓	✓		✓							
Pramparo et al., 2015	✓		✓						✓		✓		
Prandini et al., 2014	✓		✓	✓									
Segura et al., 2015	✓		✓	✓		✓						✓	
Stamova et al., 2011			✓	✓									
Talebizadeh et al., 2014 (taken from AGRE)			✓	✓							✓		
Taurines et al., 2011			✓	(ADI)									
Tian et al., 2011			✓	✓					✓		✓		
Walker et al., 2013	✓		✓	✓					✓	✓	✓		
Williams et al., 2011	✓			✓									
Yasuda et al., 2011	✓												

**Table 4B:** **Diagnostic tests part 2**.

**Paper/diagnostic test**	**Social communication questionnaire**	**Shortened CPEA regression interview**	**ICD-10**	**PARS**	**Japanese version of the Asperger's questionnaire**	**Japanese version of the autism spectrum Quotient (AQ-J)**	**Japanese versions of WAIS-III**	**PPVT**	**Inclusion criteria**	**Exclusion criteria**
Alter et al., 2011									For Probands: A negative Fragile X DNA test, impairment in language	For Probands: significant prenatal history (prematurity, 35 weeks, intraventricular hemorrhage, severe asphyxia, or cerebral palsy), serious CNS abnormality, known genetic or metabolic disorder, non-classic forms of autism were excluded, including autism with regression and Asperger's syndrome, a higher functioning form of autism where individuals have language skills within the normal range
Emanuele et al., 2010									For controls: no past or present history of any psychiatric disorder and none of them had ever taken medications for psychiatric conditions	For controls: subjects with axis-I diagnosis of first-degree relatives
Enstrom et al., 2009									ASD: children who scored above the cut-off for the ADOS modules 1 and 2 for ASD and met the criteria for autism	Children who were ill at the time of the study, or had a temperature above 98.9°F, or were prescribed anti-psychotics, or had a known medical disorder or primary diagnosis (e.g., Fragile X or Rett syndrome)
Féron et al., 2016			✓						For controls: neither presenting a neuropsychiatric disorder nor taking medication	
Ginsberg et al., 2012									Male gender; autism diagnosis by a validated psychiatric/psychologic instrument; and the availability of sufficient fresh frozen tissue available for genome-wide methylation analysis, bisulfite sequencing, and gene expression studies	Formalin-fixation of brains, brains from individuals with a medication history of medications known or suspected to have effects on methylation; gross structural abnormalities of the brain; brains from individuals with a complicated birth history and/or evidence of pre- or perinatal hypoxia; history of major head trauma; diagnosis of Rett syndrome, Fragile X syndrome, tuberous sclerosis, or other syndromic process; or any known or likely pathologic cytogenetic abnormality identified by either routine karyotyping or chromosomal microarray analysis
Hu et al., 2006								✓		
Hu et al., 2009										All females, individuals with cognitive impairment, those with known genetic or chromosomal abnormalities (e.g., Fragile X, Retts, tuberous sclerosis, chromosome 15q11-q13 duplication), those born prematurely (< 35 weeks gestation), those with diagnosed comorbid psychiatric disorders (e.g., bipolar disorder, obsessive compulsive disorder, severe anxiety)
James et al., 2014										For Case Donors: PDD-NOS, Asperger's, Rett or Fragile X For Control Donors: Previous medical history of neurologic disorders, seizures or mental retardation
Kong et al., 2013									ASD: No known genetic or syndromic disorders	For controls: chronic disease such as infectious disease, diabetes, cardiovascular disease, and developmental disorder or neurological disorder
Kuwano et al., 2011						✓	✓			For controls: serious physical or mental disorders including ASD in the past and at present
Prandini et al., 2014									ASD: meets Diagnostic and Statistical Manual of Mental Disorders, fourth edition criteria for Autistic Disorder, Asperger's Disorder, or Pervasive Developmental Disorder Not Otherwise Specified (PDD NOS); reaches the score cutoff in Autism Diagnosis Interview-Revised (ADI-R) and Autism Diagnostic Observation Schedule (ADOS); is at least 4 years old at the time of entering the research project; has at least one parent or legal guardian giving voluntary written consent for him/her to participate in the research project, and gives his/her assent when possible	For ASD: diagnosis of Rett syndrome or childhood disintegrative disorder, history of serious head injury, encephalitis or tumors, profound mental retardation (intelligence quotient < 20), and age more than 18 years
Segura et al., 2015									For ASD: Had a total IQ score above 70 For controls: no somatic or neurological disease and without medication	
Talebizadeh et al., 2014 (taken from AGRE)	✓									
Taurines et al., 2011										For controls: if had somatic or neurological disease or were taking medication
Tian et al., 2011	✓								For Autism: meeting criteria on the communication, social, and repetitive behavior domains of the ADI-R, and scoring at or above the total cutoff for autistic disorder on the ADOS module 1 or 2	
Walker et al., 2013										For controls: identifiable gastrointestinal pathology
Williams et al., 2011		✓								For controls: Developmental disturbances, including ASD
Yasuda et al., 2011				✓	✓					For healthy controls: neurological or medical conditions that could potentially affect the central nervous system, had any psychiatric diseases and/or received psychiatric medication, had first- or second-degree relatives with psychiatric disease or presented with an IQ < 70
Zhubi et al., 2014- brain samples taken from the Harvard Brain Tissue Resource Center									For Control donors: free of neurological disorders, seizures, mentalretardation, dementia	For Case Donors: Asperger's syndrome Fragile–X syndrome, RTT, pervasive developmental disorder not otherwise specified, and 15q11-q13 duplication

Additionally, demographic variability between subjects and between subjects and controls complicates analysis. Considering that ASD is 4.5 times more common in males than females, the ratio of subjects in each study is of great importance if it is meant to represent the broader ASD population. While some studies took the disproportionate male incidence into consideration in choosing their subjects, others did not. The clinical presentation of ASD also varies by age. Therefore, significant age differences between ASD subjects and controls such as those in the study by Chien (Chien et al., 2013) deserve further attention.

## Conclusion

One of the advantages of gene expression studies over whole genome sequencing studies or Enzyme-Linked Immunosorbent Assay (ELISA) based protein studies for ASD is that it allows for broad screening for unique aspects of the disorder while maintaining a level of specificity that the other modalities cannot provide. In whole genome sequencing studies, millions of base pairs are analyzed and often nothing significant is found, or the few sporadic single nucleotide polymorphisms (SNP's) or copy number variants (CNV's) lack any useful context. While admittedly, more than 100 ASD-susceptibility genes have been found, the utility of this information remains elusive. In gene expression studies, on the other hand, despite analyzing large amounts of genes, the thresholds for differences in expression enable a level of specificity and the ability to group specific genes together in order to identify specific pathways. Approximately 12,000 genes were differentially expressed between ASD compared to controls in gene expression studies since 2011. Most of studies can be subdivided by the source into three categories: brain (~3500 dysregulated genes), LCL (~5600 dysregulated genes), and GI (~2600 dysregulated genes). More specifically, in the gene expression studies of ASD surveyed, cell cycle, GI disease, immune function, and neurogenesis, were found to be the most common implicated pathways.

Most of the genes surveyed were shown to be consistently down or up-regulated across different source types in different studies. This strongly suggests that, in fact, these genes are not coincidentally higher or lower in ASD but might actually be active players in the underlying pathogenesis of the disorder.

### Future directions

Researchers might want to consider testing more than one sample source (peripheral blood, intestine, olfactory stem cell, and hair follicles) for each subject to help determine if up or downregulation is consistent amongst tissue types. In the absence of comparing across sample source types it remains unclear if differences between subjects in gene regulation are due to the different subjects or the different sample source.

Furthermore, researchers might consider including more healthy mothers of children with ASD in the gene expression studies and TD siblings in order to help isolate potential immunogenetic processes. This might help clarify why certain immune mechanisms affect only the child with ASD and not the mother or the siblings. Another idea could be to analyze the blood of daughters of women who have given birth to children with ASD in order to test whether they too have abnormal levels of certain proteins or immune markers that their healthy mothers have. If found, the abnormal levels could suggest that the healthy mother is passing something on to her daughter that would then make her more susceptible to having a child with ASD herself.

Additionally, now that certain pathways have been identified as being associated with ASD, researchers can work backwards and look for other genes involved in those pathways and test whether these specific genes play a role in ASD.

Finally, in order to help identify potential sub-groups of ASD, it might be fruitful to study correlations between subsection scores on ADOS and gene expression studies. This might unravel the heterogeneity of ASD into individual strands whose underlying pathology can be better understood at the genetic and epigenetic levels in order to develop targeted therapeutic approaches.

## Author contributions

AA drafted the article, reviewed the relevant literature, made substantial contributions to conception and design, interpreted the data and approved final version. JR drafted drafted the article, reviewed the relevant literature, made substantial contributions to conception and design, interpreted the data, revised the article critically and approved final version. PZ and MM made substantial contributions to conception and design, participated in revising it critically, interpreted the data, and approved final version. BG participated in revising it critically, interpreted the data, and approved final version.

## Funding

This study was supported by Cell-EL LTD.

### Conflict of interest statement

The authors declare that the research was conducted in the absence of any commercial or financial relationships that could be construed as a potential conflict of interest.

## Abbreviations

ADI-R: autism diagnostic interview revised
ANOVA: analysis of variance
ASD: autism spectrum disorders
asdMO: mothers having children with ASD
CNV's: copy number variants
DAVID: Database for Annotation Visualization and Integrated Discovery
DSM-5: Diagnostic and Statistical Manual of Mental Disorders Fifth Edition
ELISA: Enzyme-Linked Immunosorbent Assay
IFNγ: interferon gamma
IL: interleukin
LCL: lymphoblastoid cell lines
mRNA: messenger ribonucleic acid
MGAM: maltase glucoamylase
NOS: nitric oxide synthetase
PCs: Purkinje cells
RT-qPCR: Real-time quantitative polymerase chain reaction
SAM: significance analysis of microarrays
SNP's: single nucleotide polymorphism
TD: typically developing/developed.
